# Production of [^211^At]NaAt solution under GMP compliance for investigator-initiated clinical trial

**DOI:** 10.1186/s41181-024-00257-z

**Published:** 2024-04-15

**Authors:** Sadahiro Naka, Kazuhiro Ooe, Yoshifumi Shirakami, Kenta Kurimoto, Toshihiro Sakai, Kazuhiro Takahashi, Atsushi Toyoshima, Yang Wang, Hiromitsu Haba, Hiroki Kato, Noriyuki Tomiyama, Tadashi Watabe

**Affiliations:** 1 Department of Nuclear Medicine and Tracer Kinetics, Osaka University Graduate School of Medicine, 2-2 Yamadaoka, Suita, Osaka 565-0871 Japan; 2 Department of Pharmacy, Osaka University Hospital, 2-15 Yamadaoka, Suita, Osaka 565-0871 Japan; 3 Institute for Radiation Sciences, Osaka University, 2-4 Yamadaoka, Suita, Osaka 565-0871 Japan; 4 Hanwa Intelligent Medical Center, Hanwa Daini Senboku Hospital, 3176 Fukaikitamachi, Naka- ku, Sakai, Osaka 599-8271 Japan; 5 Advanced Clinical Research Center, Fukushima Global Medical Science Center, Fukushima Medical University, 1 Hikarigaoka, Fukushima, Fukushima 960-1295 Japan; 6 Nishina Center for Accelerator-Based Science, RIKEN, 2-1 Hirosawa, Wako, Saitama 351-0198 Japan; 7 Department of Radiology, Osaka University Graduate School of Medicine, 2-2 Yamadaoka, Suita, Osaka 565-0871 Japan

**Keywords:** Targeted alpha therapy, Astatine, [^211^At]NaAt, Automated dry distillation system, Thyroid cancer

## Abstract

**Background:**

The alpha emitter astatine-211 (^211^At) is garnering attention as a novel targeted alpha therapy for patients with refractory thyroid cancer resistant to conventional therapy using beta emitter radioiodine (^131^I). Herein, we aimed to establish a robust method for the manufacturing and quality control of [^211^At]NaAt solution for intravenous administration under the good manufacturing practice guidelines for investigational products to conduct an investigator-initiated clinical trial.

**Results:**

^211^At was separated and purified via dry distillation using irradiated Bi plates containing ^211^At obtained by the nuclear reaction of ^209^Bi(^4^He, 2n)^211^At. After purification, the ^211^At trapped in the cold trap was collected in a reaction vessel using 15 mL recovery solution (1% ascorbic acid and 2.3% sodium hydrogen carbonate). After stirring the ^211^At solution for 1 h inside a closed system, the reaction solution was passed through a sterile 0.22 μm filter placed in a Grade A controlled area and collected in a product vial to prepare the [^211^At]NaAt solution. According to the 3-lot tests, decay collected radioactivity and radiochemical yield of [^211^At]NaAt were 78.8 ± 6.0 MBq and 40 ± 3%, respectively. The radiochemical purity of [^211^At]At^−^ obtained via ion-pair chromatography at the end of synthesis (EOS) was 97 ± 1%, and remained > 96% 6 h after EOS; it was detected at a retention time (RT) 3.2–3.3 min + RT of I^−^. LC-MS analysis indicated that this principal peak corresponded with an astatide ion (m/z = 210.988046). In gamma-ray spectrometry, the ^211^At-related peaks were identified (X-ray: 76.9, 79.3, 89.3, 89.8, and 92.3 keV; γ-ray: 569.7 and 687.0 keV), whereas the peak at 245.31 keV derived from ^210^At was not detected during the 22 h continuous measurement. The target material, Bi, was below the 9 ng/mL detection limit in all lots of the finished product. The pH of the [^211^At]NaAt solution was 7.9–8.6; the concentration of ascorbic acid was 9–10 mg/mL. Other quality control tests, including endotoxin and sterility tests, confirmed that the [^211^At]NaAt solution met all quality standards.

**Conclusions:**

We successfully established a stable method of [^211^At]NaAt solution that can be administered to humans intravenously as an investigational product.

## Background

In the treatment of differentiated thyroid cancer, radionuclide therapy with radioactive iodine ([^131^I]NaI), which emits beta-rays, is the standard treatment for patients with metastatic lesions after surgery. However, some patients show insufficient therapeutic effects on iodine-avid lesions despite receiving multiple doses of this treatment. Therefore, the development of a more effective treatment using alpha-emitter is a promising approach for patients with refractory thyroid cancer.

The alpha-emitter astatine (^211^At) belongs to the halogen family and its properties are similar to those of iodine, a homologous element. We previously reported that ^211^At-sodium astatide ([^211^At]NaAt) is taken up by differentiated thyroid cancer cells via the sodium-iodide symporter (NIS), similar to iodine (Watabe et al. 2019, 2022). A high therapeutic effect, with minimal damage to the surrounding normal tissue, can be achieved through selective accumulation in the tumor lesion because alpha rays have a short range and transfer high energy to induce deoxyribonucleic acid (DNA) double-strand breaks (McDevitt et al. 2018). In a preclinical study using mouse xenograft models with NIS-expressing thyroid cancer, we confirmed the high accumulation of [^211^At]NaAt in K1-NIS tumors, showing better dose-dependent antitumor effects than [^131^I]NaI (Watabe et al. 2019, 2022). Furthermore, no critical toxicity was observed in a toxicity study in mice following a single intravenous administration at doses up to 50 MBq/kg body weight (Liu et al. 2020; Watabe et al. 2021b).

Consequently, an investigator-initiated clinical trial has been conducted at the Osaka University Hospital, using [^211^At]NaAt in patients with refractory differentiated thyroid cancer who failed to respond to standard treatment using radioiodine therapy ([^131^I]NaI) (ClinicalTrials.gov Identifier: NCT05275946). This study is a phase I clinical trial (first in human) to assess the safety, pharmacokinetics, absorbed dose, and efficacy of [^211^At]NaAt after a single administration. It is conducted following the good clinical practice and safety handling guidelines of the Japanese Society of Nuclear Medicine (Watabe et al. 2021a; Hertz et al. 2022). In this study, we aim to establish a method for the manufacturing and quality control of a stable supply of [^211^At]NaAt solution for intravenous administration, under good manufacturing practices (GMP) for investigational products for this clinical trial at the short-lived radioactive drug manufacturing facility of Osaka University Hospital, and further confirmed them via 3-lot tests.

## Methods

### Irradiated Bi target containing [^211^At]at

**Fig. 1:**
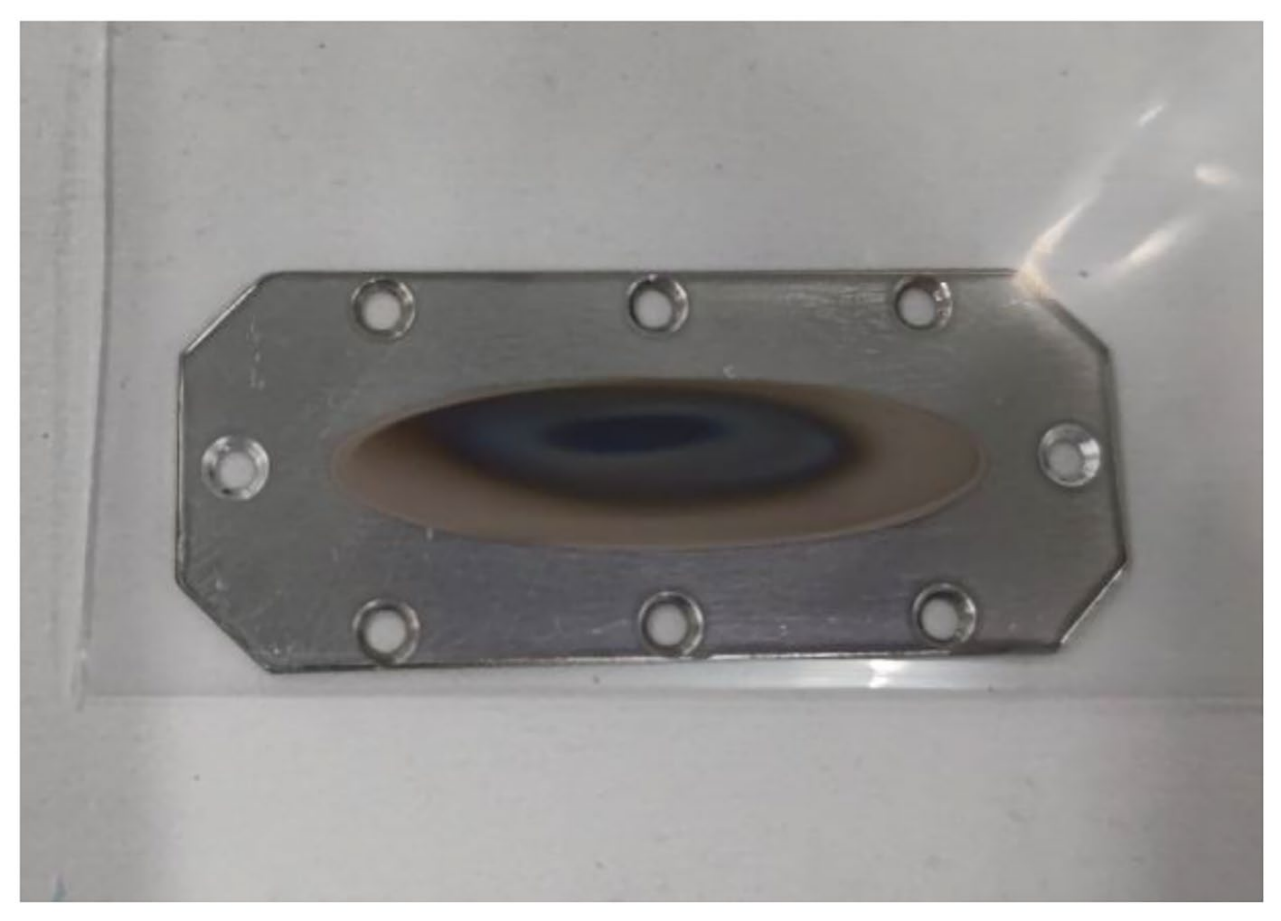
Irradiated Bi target containing [^211^At]astatine. After irradiation, the target was sealed in a polyethylene bag with a zipper for storage and transport

We procured ^211^At in the form of an irradiated Bi plate containing ^211^At (Fig. 1), which was obtained by the nuclear reaction of ^209^Bi(^4^He, 2n)^211^At using the K70 AVF cyclotron of RIKEN RI Beam Factory, Saitama, Japan (Sato et al. 2017). The ^211^At contained in the Bi plate was purified using the dry distillation method at the Osaka University Hospital.

### Reagents for [^211^At]NaAt synthesis

Biotechnology-grade L(+)-ascorbic acid, used as a reducing agent, was purchased from Nacalai Tesque (Kyoto, Japan). Japanese Pharmacopoeia grade sodium hydrogen carbonate (7%) and water were purchased from Otsuka Pharmaceuticals (Tokyo, Japan). Molecular sieves 3 A 1/16 (FUJIFILM Wako Pure Chemical, Osaka, Japan), nickel plates (0.20 × 300 × 300 mm; Nilaco, Tokyo, Japan), helium gas (> 99.9999% purity; Taiyo Nippon Sanso, Tokyo, Japan), and oxygen gas (general grade; Iwatani Fine Gas, Hyogo, Japan) were used for [^211^At]NaAt synthesis.

### Separation and purification of ^211^At

**Fig. 2:**
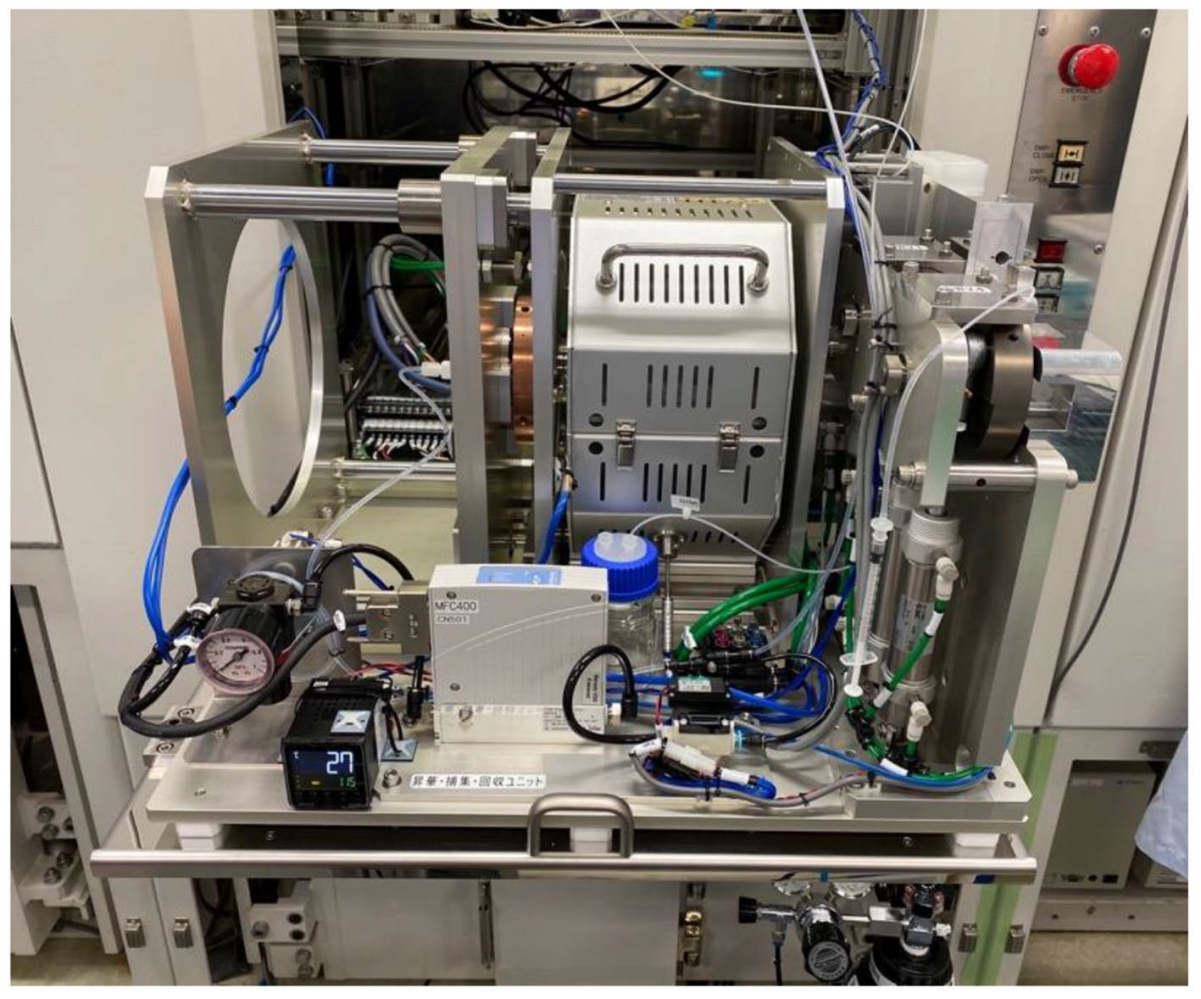
^211^At automated distillation system COSMiC-Mini VTRSC2. This synthesizer was installed in a hot cell in a Grade B controlled area in Osaka University Hospital

**Fig. 3:**
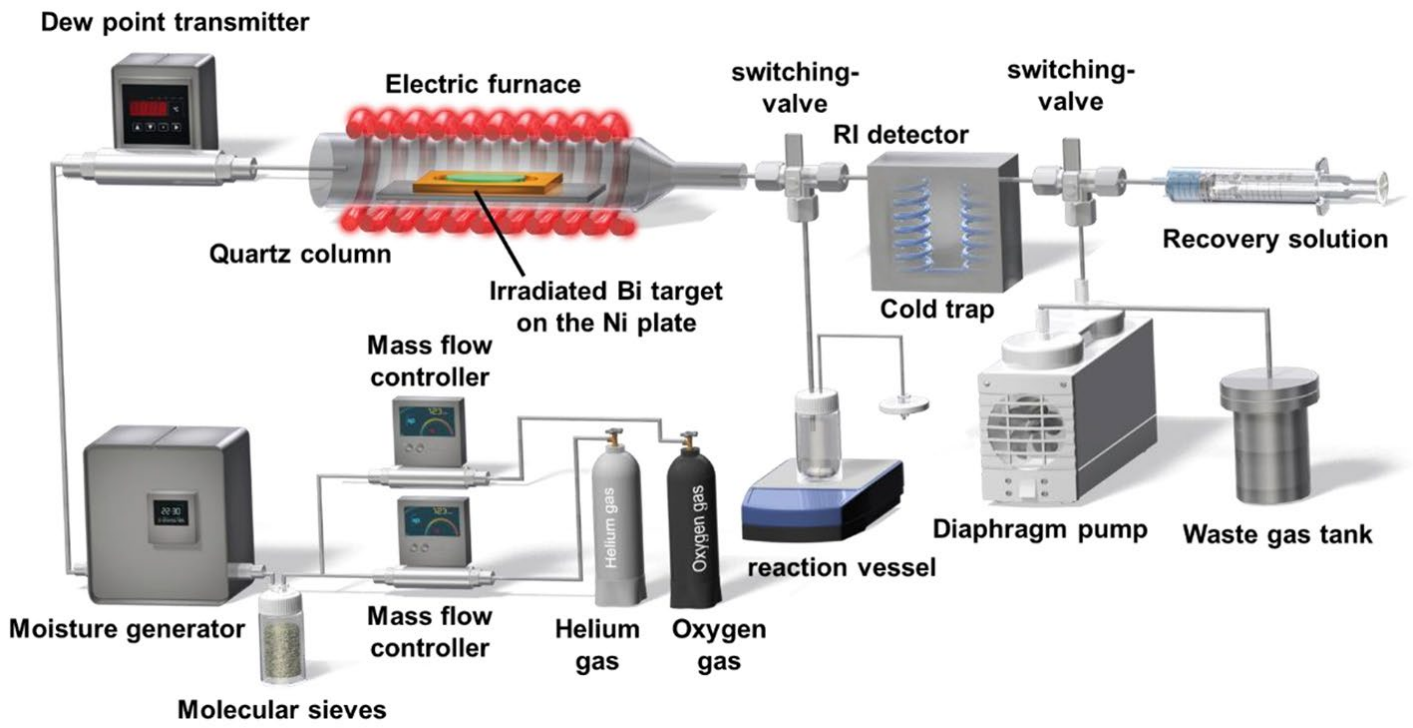
Synthesis diagram of [^211^At]NaAt by dry distillation system

We separated and purified ^211^At from the irradiated Bi target through dry distillation, using the automated dry distillation system COSMiC-Mini VTRSC2 (Nihon Mediphysics Business Support, Hyogo, Japan), which was installed in a hot cell in a Grade B controlled area (Figs. 2 and 3). The first step was to remove the residual water inside the system. Two stacked Ni plates (0.20 × 40 × 75 mm) were placed in a quartz glass holder and set inside a quartz column in an electric furnace. After closing the electric furnace, the temperature was raised to 850 °C while He gas flowing at the rate of 20 mL/min was passed through the molecular sieves; the system was maintained at these conditions for 30 min. After sufficient cooling, the electric furnace was opened, the irradiated Bi target was quickly placed on the Ni plate, and the furnace was closed. Irradiated Bi targets that passed the acceptance tests (irradiation record, radioactivity, appearance, and radionuclidic identity) were used for [^211^At]NaAt synthesis.

Subsequently, 1 mL of water was added to a dedicated vial connected upstream of the electric furnace, and the moisture content of the carrier gas was adjusted. A dew point instrument (DMT143L; Vaisala, Helsinki, Finland) was used to monitor the supplied moisture content. The temperature of the dedicated vial was adjusted using a temperature controller so that the dew-point temperature ranged from − 6 to -7 °C (moisture content: 3400 to 3700 ppm) while a mixture of He (6 mL/min) and O_2_ (10 mL/min) gas flowed through the molecular sieves. In addition, a cold trap (-3 °C) to capture the separated ^211^At, and the two heaters (115 °C) installed between the electric furnace and the cold trap to prevent the adsorption of ^211^At, were turned on during conditioning by the carrier gas flow for 20 min to stabilize the moisture content in the quartz column.

Once all the set-points reached the desired conditions, the electric furnace (850 °C) was turned on to commence ^211^At separation. When the temperature of the electric furnace reached 850 °C and the trapped radioactivity in the cold trap was constant, the gas supply and the heating of the electric furnace were stopped.

### Production of [^211^At]NaAt solution

After the switching-valve was switched, ^211^At that had trapped in the cold trap was collected in a reaction vessel (20 mL sealable sterile vial; Mita Rika Kogyo, Hyogo, Japan) in the reverse direction, using 15 mL of the recovery solution (1% ascorbic acid and 2.3% sodium hydrogen carbonate). The recovery solution was prepared by adding equal amounts of 3% aqueous ascorbic acid solution, 7% sodium hydrogen carbonate, and water. The ^211^At solution collected in the reaction vessel was reacted with ascorbic acid to convert the astatine oxides to ^211^At^−^; thus, the reaction was carried out in a closed system for 1 h, which is a sufficient reaction time, while avoiding contamination with air from outside the system. Lastly, the reaction solution was passed through a sterile 0.22-µm Millex GV filter (Merck Millipore, Burlington, MA) placed in a Grade A controlled area (clean booth) and collected in a product vial (20 mL sealable sterile vial) with 20 mL/min of He gas.

### Microbiological and airborne particulate evaluation

Environmental monitoring was performed to ensure a Grade A controlled area in the filter sterilization area (clean booth in a hot cell) and dispensing operation area (clean bench) for [^211^At]NaAt solution production, and a Grade B controlled area in the synthesis room (one point in the workflow line), and on workers (gowns and gloves), following the Japanese Pharmacopoeia guidelines.

### Quality tests for [^211^At]NaAt

The quality parameters and acceptance criteria for [^211^]NaAt are listed in Table 1. The radioactivity of [^211^At]NaAt and its half-life were measured using a dose calibrator (Capintec, Florham Park, NJ) calibrated with ^211^At in advance. Calibration was conducted using a ^211^At source, whose radioactivity was determined using a Ge semiconductor detector (BE2020; Mirion Technologies Canberra, Meriden, CT). The radiochemical purity and identity of [^211^At]At^−^ were measured using a Prominence high-performance liquid chromatography (HPLC) system, including the CBM-20 A communications bus, SPD-20 A UV/VIS detector, CTO-20AC column oven, SIL-20ACHT autosampler, LC-20AD pump, and DGU-20A_5R_ degasser (Shimadzu, Kyoto, Japan) and a Gabi-Nova radioactivity detector (Raytest, Straubenhardt, Germany). The analysis column used was a COSMOSIL 5C_18_-MS-II (150 mm × 4.6 mm; Nacalai Tesque) with a mobile phase of 20 mmol/L tetra-n-butylammonium chloride/acetonitrile (70/30). The flow rate and the column oven temperature were set to 1.0 mL/min and 25 °C, respectively. In addition, no natural isotope of At exists; therefore, I^−^ (NaI, FUJIFILM Wako Pure Chemical), which is the most homologous compound to At, was used as a reference standard for the HPLC system suitability test and identification of [^211^At]At^−^ at wavelength 254 nm. The radionuclidic identity (including the acceptance test of the target) and radionuclidic purity were analyzed using a Ge detector calibrated for energy and peak efficiency with ^137^Cs and ^133^Ba standard sources. As a system check of the Ge detector before ^211^At analysis, the ^133^Ba standard source was measured. The region of interests was set near 81.0 and 383.9 keV, and the central energy was maintained within ± 1 keV of these energy values. The endotoxin test was performed using the turbidimetric technique with a Toxinometer® ET-6000 (FUJIFILM Wako Pure Chemical) in accordance with the Japanese Pharmacopoeia guidelines. Since the maximum dosage was 15 mL, the acceptance criteria was 10.0 EU/mL, and the [^211^At]NaAt solution was diluted 20-fold. The pH was measured using the Benchtop pH Meter F-72 combined with the Micro ToupH Electrode (HORIBA, Kyoto, Japan), for low-volume samples, calibrated with standard pH buffer solutions. Sterility tests were performed using two different media via direct inoculation. In this process, 0.5 mL of [^211^At]NaAt was added to a 100 mL bottle of fluid thioglycollate medium and soybean casein digest medium (Merck), respectively, and the growth of micro-organisms after incubation at 32.5 °C and 22.5 °C, respectively, was checked on day 14 of the test. In addition, a method suitability test for sterility tests of the [^211^At]NaAt solution was performed by KANKYO EISEI YAKUHIN CO., LTD., following the Japanese Pharmacopoeia guidelines, before the 3-lot tests. The concentration of ascorbic acid to be added as a reducing agent was measured using the RQflex® 20 reflectometer and Reflectoquont® ascorbic acid test strip (Merck) on a [^211^At]NaAt solution diluted 200-fold with water. A filter integrity test was conducted to evaluate the performance of the sterilized filter. The bubble point value of the aqueous solution recommended by the supplier (> 345 kPa) was used as the acceptance criterion and was measured using a filter integrity tester UG-FT02 (Universal Giken, Kanagawa, Japan).

**Table 1:** Test items and acceptance criteria of quality tests for [^211^At]NaAt solution

Test items	Acceptance criteria	Test method
Appearance	Clear and colorless to light yellow	Visual inspection
Particle	None	Visual inspection
Identity of [^211^At]NaAt	RT of NaI + 2.5–+ 3.5 min	HPLC (Radioactivity and UV/VIS detector)
Half-life	6.8–7.6 h	Dose-calibrator
Concentration of radioactivity (at EOS)	≧ 4.0 MBq/mL	Dose-calibrator
Radionuclidic identity	Exhibits the peaks at 76.9 keV, 79.3 keV and 687.0 keV	γ-ray spectrometry with Ge semiconductor detector
Radiochemical purity (at EOS)	≧ 85%	HPLC (Radioactivity detector)
Contamination from other radionuclide	Exhibits no peak except comes from ^211^At	γ-ray spectrometry with Ge semiconductor detector
pH	7.0–9.0	glass electrode method
Sterility	Sterile	Direct inoculation
Endotoxin	< 10.0 EU/mL	Turbidimetric technique
Concentration of ascorbic acid	≧ 6 mg/mL	Reflection photometer
Filter integrity test	≧ 345 kPa	Bubble point method

EOS, end of synthesis; RT, retention time

In addition to these routine quality tests, 3-lot tests on the [^211^At]NaAt solution were performed to assess residual Bi using inductively coupled plasma-mass spectrometry (ICP-MS 7800; Agilent, Santa Clara, CA), ^210^At contamination through long-term measurement (approximately 22 h) using the Ge detector, and osmotic pressure via the freezing point effect method (Osmometer OM815; VOGEL, Fernwald, Germany). For measuring residual Bi using ICP-MS, the [^211^At]NaAt solution was diluted 10,000-fold using 0.32 M HNO_3_ which was prepared by diluting concentrated nitric acid (FUJIFILM Wako Pure Chemical) with Milli-Q water (Merck). Thallium was used as an internal standard for residual Bi measurement using ICP-MS. The calibration curve for the Bi concentration was obtained by measuring metal standard solutions prepared from 100 mg/L of Al, Bi, Pb, and Ni in 1 M HNO_3_ (ICP standard solution A; Kanto Chemical, Tokyo, Japan) diluted with 0.32 M HNO_3_. Each sample was measured ten times.

### Liquid chromatography-mass spectrometry (LC-MS) analysis for quantification of ^211^At

To confirm that the principal peak obtained in the radiochemical purity measurement was that of [^211^At]At^−^, LC-MS analysis was performed on the radioactive solution obtained using the same procedure as that in the 3-lot tests. This analysis was performed using an LCMS-9030 system including the CBM-40 system controller, SPD-M40 photodiode array detector, CTO-20AC column oven, SIL-20ACHT autosampler, LC-20AD pump and DGU-405 degasser, Shimadzu, Kyoto, Japan and a radioactivity detector (Gabi-Nova) connected in front of the MS introduction. The Shodex HILICpak VT-50 2D (Resonac, Tokyo, Japan) 150 mm × 2.0 mm analysis column was used with a mobile phase containing 50 mM ammonium formate/acetonitrile (20/80). The flow rate and the column oven were set at 0.3 mL/min and 40 °C, respectively.

## Results

### Production and quality test of [^211^At]NaAt solution

Through the acceptance test for the irradiated Bi plate containing ^211^At, we determined that the delivered ^211^At radioactivity in each of the 3-lots (approximately 250 MBq) did not exceed the upper limit of the facility (2.5 GBq/day), and its physical condition was solid. The irradiation energy (28.1 MeV) for the Bi target was less than 28.6 MeV, which was below the threshold energy for the ^209^Bi(^4^He, 3n)^210^At nuclear reaction. After the target was removed, the presence of ^211^At in the irradiated Bi target was confirmed with the Ge detector, which detected X-rays of 76.9 and 79.3 keV and a γ-ray of 687.0 keV from ^211^At.

**Fig. 4:**
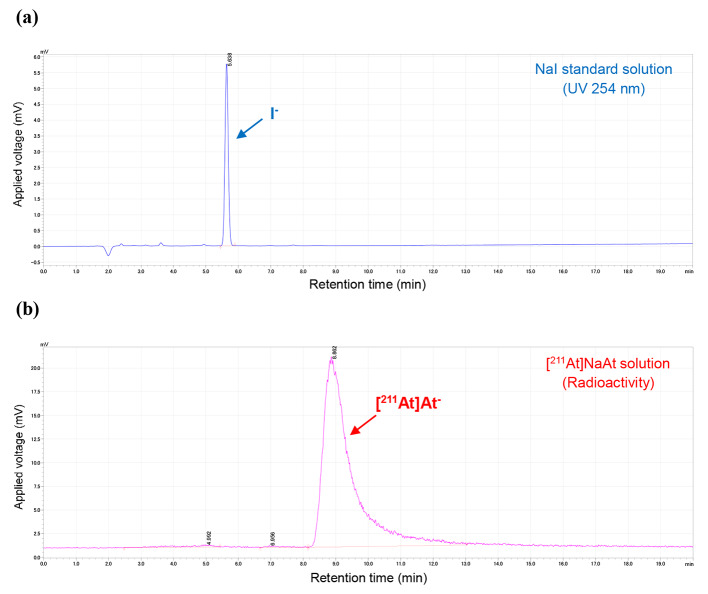
Typical chromatogram of NaI standard solution and [^211^At]NaAt solution. **(a)** I^−^ was measured at 254 nm and its RT was approximately 5.6 min. **(b)** [^211^At]At^−^ was measured by a radioactivity detector using a 250 µL semi-preparative sample loop to ensure sufficient peak area and its RT was approximately 8.9 min. Unknown radiochemical impurities appeared at approximately 5.0 and 7.0 min

**Fig. 5:**
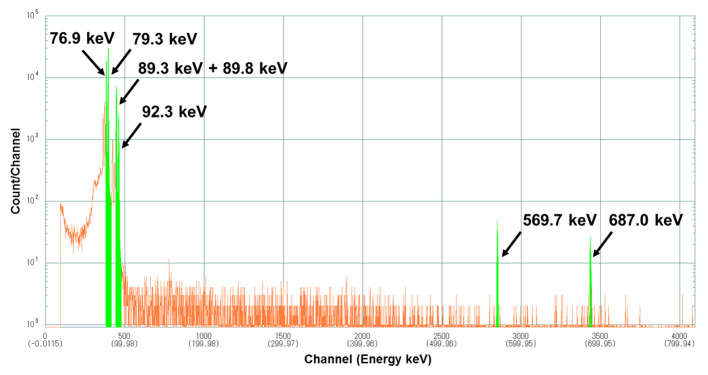
Typical γ-ray spectrum of [^211^At]NaAt solution. We detected X radiation of 76.9, 79.3, 89.3, 89.8 and 92.3 keV derived from ^211^Po, a daughter nuclide of ^211^At, and γ radiation of 569.7 and 687.0 keV derived from ^211^Po and ^211^At, respectively

The production of [^211^At]NaAt solution was achieved within 3 h from the setting of the irradiated Bi target in the automated dry distillation system; the radioactivity and the radiochemical yield were 78.8 ± 6.0 MBq and 40 ± 3%, respectively, at the end of synthesis (EOS). The radioactivity peak of [^211^At]At^−^ (8.9 min) was detected at 3.2–3.3 min + the retention time (RT) of I^−^ (5.6–5.7 min); the radiochemical purity of [^211^At]At^−^ at EOS was 97 ± 1% (Fig. 4). Furthermore, the radiochemical purity was greater than 95% at 6 h after EOS. In the γ-ray spectrum of the [^211^At]NaAt solution, no peaks other than the ^211^At-derived peak (X-rays of 76.9, 79.3, 89.3, 89.8, and 92.3 keV were derived from ^211^Po, a daughter nuclide of ^211^At, and γ rays of 569.7 and 687.0 keV were derived from ^211^Po and ^211^At, respectively) were identified (Fig. 5). Furthermore, the peak at 245.31 keV derived from ^210^At (branch ratio 79%) was not detected in the 22 h continuous measurement (data not shown). The detection limit for ^210^At in the 22 h continuous measurement was calculated previously (Cooper et al. 1970), and the radioactivity ratio for ^210^At/^211^At was estimated to be less than 7 × 10^− 4^%. The endotoxin test results were below the lower limit (0.1 EU/mL) of the calibration curve, and sterility tests showed no growth of microorganisms in any of the lots. In the method suitability test, the growth of microorganisms was visually confirmed in the presence of the [^211^At]NaAt solution and the positive control as well, for all six strains of microorganisms listed in the Japanese Pharmacopoeia (*Bacillus subtilis*, *Candida albicans*, and *Aspergillus brasiliensis* in soybean casein digest medium; *Pseudomonas aeruginosa*, *Staphylococcus aureus*, and *Clostridium sporogenes* in fluid thioglycollate medium) within 3 or 5 d. The pH of the [^211^At]NaAt solution was 7.9 to 8.6, the concentration of ascorbic acid was 9–10 mg/mL, and the bubble-point value for the filter integrity test was 411–424 kPa. All quality test results for the 3-lot tests are listed in Table 2.

**Table 2:** Results of quality test for [^211^At]NaAt solution

Test items	Acceptance criteria	Lot No.1	Lot No.2	Lot No.3
Radioactivity (MBq)	-	81.6	72.0	82.9
Appearance	Clear and colorless to light yellow	Clear and colorless	Clear and colorless	Clear and colorless
Particle	None	None	None	None
Identity of [^211^At]NaAt (min)	RT of NaI + 2.5–+ 3.5	+ 3.2	+ 3.3	+ 3.3
Half-life (hr)	6.8–7.6	7.3	7.3	7.2
Concentration of radioactivity (at EOS) (MBq/mL)	≧ 4.0	5.8	5.3	5.9
Radionuclidic identity	Exhibits the peaks at 76.9 keV, 79.3 keV and 687.0 keV	Exhibits the peaks at 76.9 keV, 79.3 keV and 687.0 keV	Exhibits the peaks at 76.9 keV, 79.3 keV and 687.0 keV	Exhibits the peaks at 76.9 keV, 79.3 keV and 687.0 keV
Radiochemical purity (at EOS) (%)	≧ 85	98	96	97
Contamination from other radionuclide	Exhibits no peak except comes from ^211^At	Exhibits no peak except comes from ^211^At	Exhibits no peak except comes from ^211^At	Exhibits no peak except comes from ^211^At
pH	7.0–9.0	8.6	8.5	7.9
Sterility	Sterile	Sterile	Sterile	Sterile
Endotoxin (EU/mL)	< 10.0	< 2.0	< 2.0	< 2.0
Concentration of ascorbic acid (mg/mL)	≧ 6	10	10	9
Filter integrity test (kPa)	≧ 345	411	424	419

EOS, end of synthesis; RT, retention time

Regarding the additional parameters, the measurement of residual Bi via ICP-MS revealed that the Bi levels were below the detection limit for all lots of [^211^At]NaAt solution. The calculated detection limit of Bi was approximately 0.9 ng/L, based on the average Bi counts in the blank (0.32 mol/L HNO_3_) solution and the calibration curve for Bi concentration. When the [^211^At]NaAt solution was diluted 10,000-fold, the concentration of Bi in the [^211^At]NaAt sample was less than 9 ng/mL. The average osmolality was 488 mOsm/L (osmotic pressure ratio = 1.7).

### Microbiological and airborne particulate evaluation

The results of environmental monitoring of each area showed that the number of airborne particulates in the Grade A controlled area (clean bench and booth) ranged from 2 to 190 /m^3^ above 0.5 μm (maximum number of particulates: 3,520 /m^3^) to 0–4 /m^3^ above 5.0 μm (maximum number: 20 /m^3^); in the Grade B controlled area (synthesis room), the number of airborne particulates ranged from 2,554 to 5,005 /m^3^ above 0.5 μm (maximum number: 352,000 /m^3^) to 92–134 /m^3^ above 5.0 μm (maximum number: 2,900 /m^3^). No microorganisms were detected in any of the lots for the airborne micro-organisms (clean bench and booth; settle plate, synthesis room; air sampling) and the surfaces (clean bench and booth, synthesis room, left gloves, right gloves, right arm, and forehead).

### LC-MS analysis for quantification of ^211^At

**Fig. 6:**
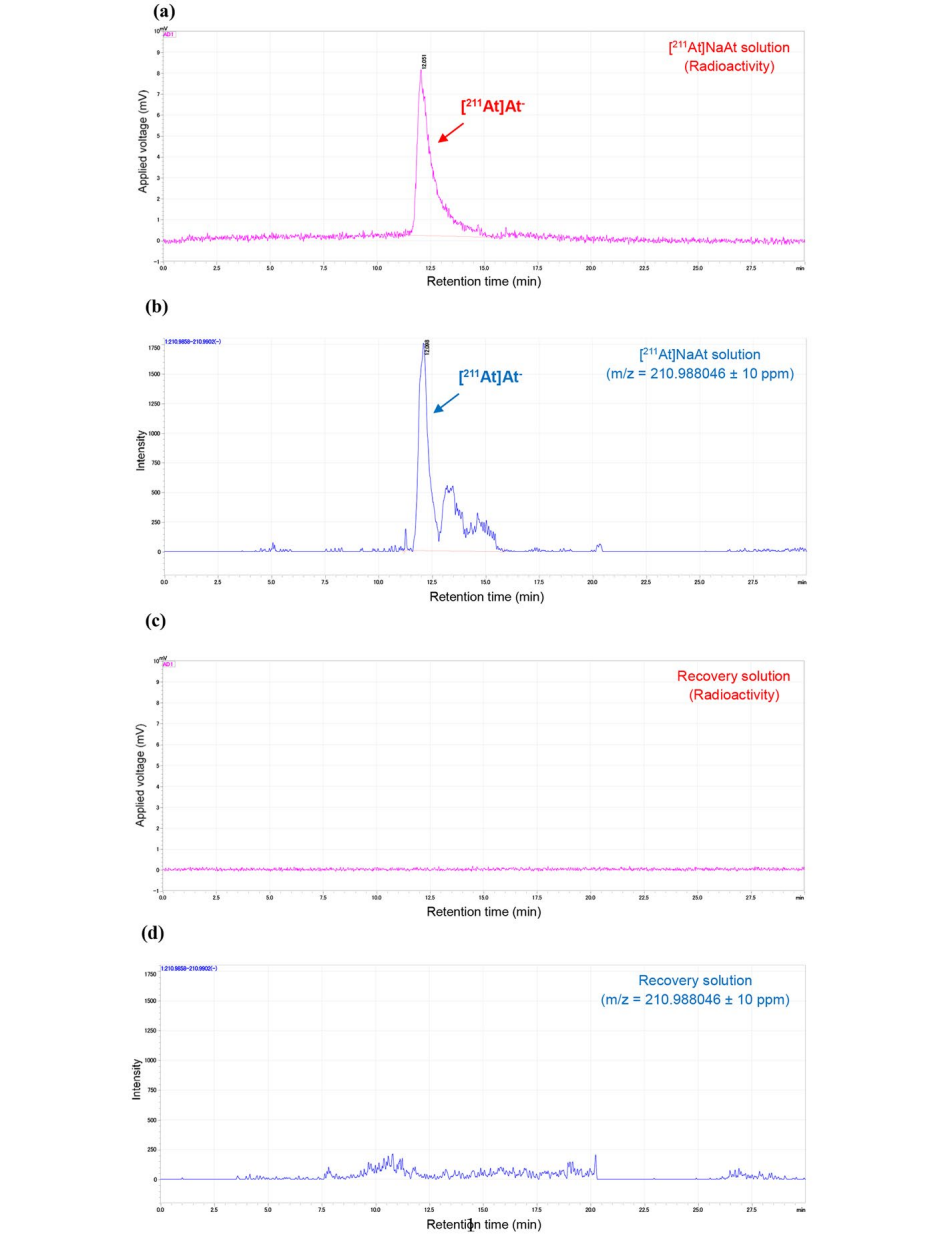
Typical HPLC chromatograms. **(a)** Typical radioactivity chromatograms of [^211^At]NaAt. **(b)** Typical mass spectrum (m/z = 210.988046 ± 10 ppm) chromatograms of [^211^At]NaAt. **(c)** The radioactivity chromatograms of recovery solution (1% ascorbic acid and 2.3% sodium hydrogen carbonate). **(d)** The mass spectrum chromatograms of recovery solution

**Fig. 7:**
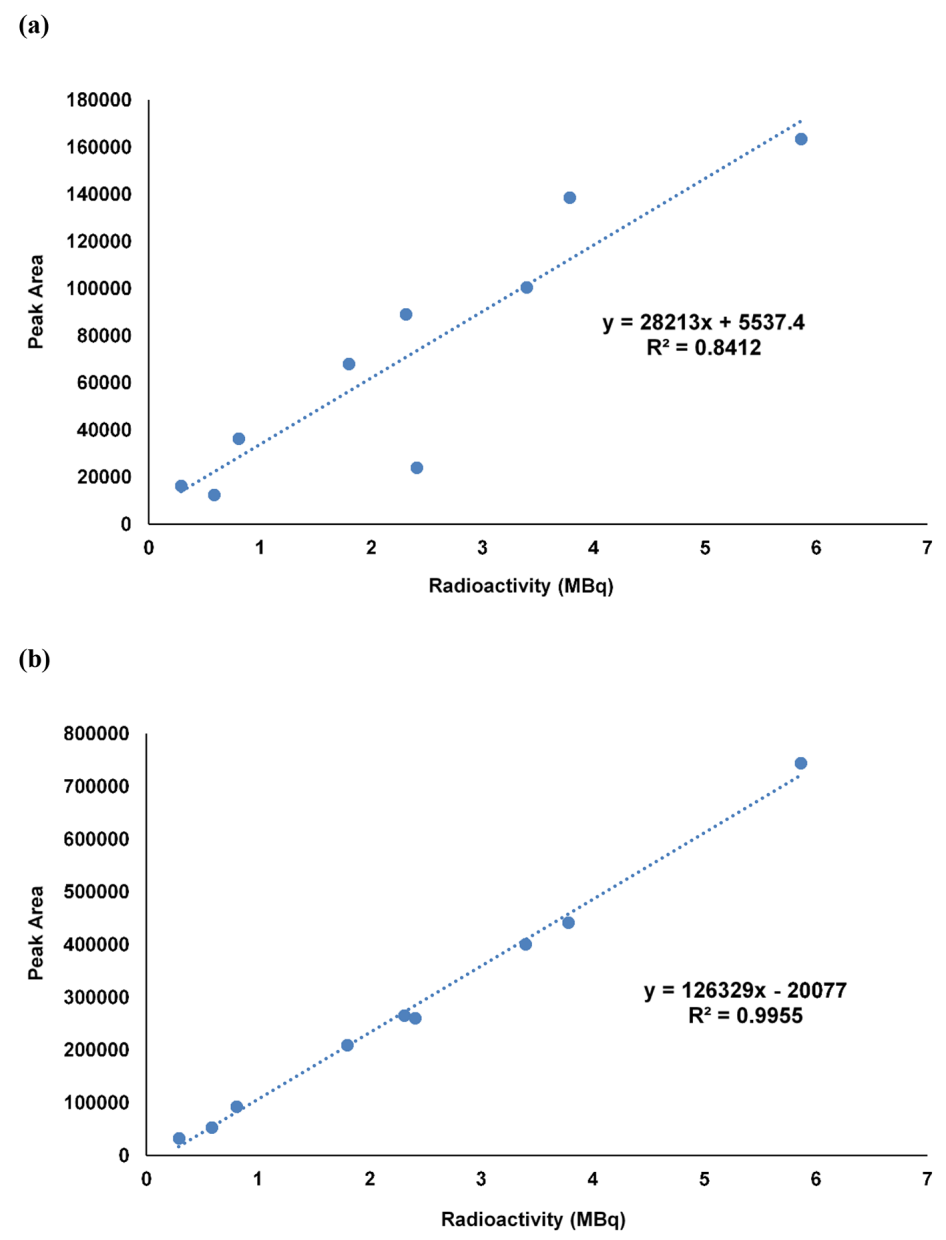
Correlation between the injection radioactivity and each peak area. **(a)** The peak area of mass spectrum (m/z = 210.988046 ± 10 ppm). **(b)** The peak area of [^211^At]NaAt radioactivity

The atomic mass ([M]) of ^211^At is 210.987496 (Audi et al. 2003), and a peak search conducted at 210.9880 ± 10 ppm (210.9858–210.9902), calculated from the negative mode measurement as [M]^−^ (mass of an electron = 0.00055), confirmed a peak at the same RT as that of [^211^At]At^−^ detected using a radioactivity detector (Fig. 6). Furthermore, the obtained peak areas showed a high correlation with the injected radioactivity (R^2^ = 0.84) (Fig. 7). In contrast, this peak was not observed in the recovery solution (1% ascorbic acid and 2.3% sodium hydrogen carbonate) (Fig. 6).

## Discussion

In this study, we successfully produced [^211^At]NaAt solutions that can be used for an investigator-initiated clinical trial. The initial dose in the clinical trial was 1.25 MBq/kg, and we confirmed that the radioactivity of the [^211^At]NaAt solution produced in this study satisfied this requirement. The protocol for this clinical trial involved gradually increasing the dose to 10 MBq/kg in the future; however, since an average radiochemical yield of 40% was obtained stably, it could be possible to increase radioactivity by increasing the starting radioactivity (below 2.5 GBq the upper limit for the facility). We had already performed scale-up manufacturing with even higher radioactivity (600 MBq) after the 3-lot tests and confirmed that we can supply the clinical requirement at about the same yield.

It has been reported that after separation, the chemical form of ^211^At is unclear (Teze et al. 2017; Appelman et al. 1961), and is assumed to exist in various chemical forms. However, we reported the method for the production of [^211^At]NaAt solution (Watabe et al. 2019), which becomes [^211^At]At^−^ by adding ascorbic acid as a reducing agent and sodium hydrogen carbonate for pH adjustment, followed by mixing with stirring for 1 h. In this study, [^211^At]At^−^ was obtained with high radiochemical purity (97 ± 1%) at EOS using HPLC. Nonclinical studies have confirmed that ascorbic acid exists stably as ^211^At^−^ when added at more than 6 mg/mL. Therefore, the acceptance criterion was set at this concentration, and the preparation amount was set at 10 mg/mL in consideration of the degradation of ascorbic acid during the manufacturing process. In our preliminary experiments, we observed the reaction over 24 h after initiating the reaction. The results showed that the highest radiochemical purity was obtained at 1 h. Radiological impurities appeared at approximately 5.0 and 7.0 min in the radio-HPLC analysis. Their chemical forms are thought to be oxides such as AtO^−^ and AtO_2_^−^; however, they have not been identified yet.

Both in vitro and in vivo experiments using [^211^At]At^−^ have been reported (Carlin et al. 2003; Spetz et al. 2013), wherein [^211^At]At^−^ was separated via dry distillation and dissolved in either phosphate-buffered saline alone or with sodium sulfite as a reducing agent; however, identification using HPLC or other methods was not performed. Furthermore, Oscar et al. deduced the obtained peak as [^211^At]At^−^ on the grounds of the increase in the abundance ratio owing to the use of a reducing agent and nearly the same RT of [^131^I]NaI in the analysis using reversed-phase HPLC (Pozzi and Zalutsky 2007). In this study, ion-pair chromatography, which has been widely used as an analytical method for I^−^ estimation, was applied for quality control testing of [^211^At]At^−^. HPLC for [^211^At]At^−^ analysis was performed using ion-pair chromatography with a reversed-phase chromatography column and tetra-n-butylammonium chloride as the eluent. Since there is no stable isotope for At, the homologous compound sodium iodide was used as a similar reference standard to check the HPLC system and to test the radiochemical purity and identity of [^211^At]At^−^. This was set as the condition for the HPLC analysis of [^211^At]NaAt produced in previous nonclinical studies (Watabe et al. 2019, 2021a, 2022; Liu et al. 2020), and based on other analyses (radio thin layer chromatography and cellulose membrane electrophoresis) results obtained by our group, it was estimated that [^211^At]At^−^ represented the principal peak detected approximately 3 min after the RT of I^−^ (data not shown). In addition, we validated the analytical method for measuring radiochemical purity using HPLC. Thus, we confirmed that the specificity (absence of significant interfering peaks during the retention time of [^211^At ]NaAt), linearity (R^2^ = 0.998), detection limit (17.5 kBq/mL), and repeatability in three injections (repeatability standard deviation = 8%) meet our specifications. Furthermore, in this study, to confirm that this peak indicated [^211^At]At^−^, time-of-flight LC-MS (TOF-LC-MS) analysis was performed against the main peak of radioactivity, and the peak was found to be consistent with the theoretical mass. The peak intensity was proportional to the radioactivity and showed a high correlation, suggesting that this peak was likely [^211^At]At^−^. Although the estimation of [^211^At]At^−^ using LC-MS has been challenging in previous studies (Appelman et al. 1966; Golovkov et al. 1980), our results demonstrate that radiopharmaceuticals, even in a femto-molar range, can be identified using the latest LC-MS, especially TOF-LC-MS, which can measure exact mass.

Radionuclidic identity and contamination from other radionuclides were analyzed using the Ge detector, and no peaks other than the ^211^At-derived peak were identified. The ^211^At-related peaks also include those derived from ^211^Po, a daughter nuclide of ^211^At that stays in the same place as ^211^At and emits alpha particles, similar to ^211^At, to exert therapeutic effects. This time, we measured only the energies up to 800.0 keV to clearly separate X-ray peaks with close energy, such as those with 76.9 and 79.3 keV. The existence of ^211^Po was confirmed using gamma rays of 569.7 keV. Further, lead-derived X-rays were detected because a lead block was used to shield the Ge detector. This was excluded from the results because it was not a sample-derived peak. Furthermore, ^210^At can decayed into highly toxic ^210^Po as a daughter nuclide (Henriksen et al. 2001), however, at lower beam energies under threshold value (28.6 MeV), ^210^At cannot be produced by the nuclear reaction of ^209^Bi(^4^He, 3n)^210^At. In this study, the Bi target was irradiated at less than 28.6 MeV, as per the acceptance test of the raw material. In addition, the 3-lot tests ensured that the radioactivity ratio for ^210^At/^211^At was less than 7 × 10^− 4^% during the long-term measurement of [^211^]NaAt solution. Based on this result, we concluded that the radionuclidic purity of the [^211^At]NaAt solution produced in this study was sufficiently high for clinical use. In this study, although we confirmed non-contamination of other nuclides including ^210^At in the test item regarding contamination from other radionuclides, we will consider adding a test item specifically related to ^210^At quantification. In addition, although Bi, the target material, was not included in the classification requiring quality control in the guideline for elemental impurities in ICH-Q3D, the level of Bi contamination measured via ICP-MS was less than 9 ng/mL (less than 135 ng for 15 mL [^211^At]NaAt, which was the maximum dosage), and was confirmed to be sufficiently less compared with the classified homologous elements As (15 µg/day) and Sb (90 µg/day).

In this study, the sterility of the [^211^At]NaAt solution was ensured through environmental monitoring during manufacturing (surface and airborne microbiological particulates), integrity testing of the sterilization filter, and sterility testing. For environmental monitoring, the grade specified for cleanrooms in the Japanese Pharmacopoeia was guaranteed, and the bubble point value for the sterilization filter performance was confirmed to pass the acceptance criteria. In addition, the results of the sterility test showed that no growth of bacteria was observed in any of the lots, while the lack of antimicrobial activity in the [^211^At]NaAt solution was confirmed by a method suitability test, which was judged to be appropriate at this time. Furthermore, because all other quality specifications were met, the [^211^At]NaAt solution was ascertained to be safe enough as an injectable solution for intravenous administration.

The platform technology has been established for the GMP manufacturing of the investigational product [^211^At]NaAt. Furthermore, this technology will be useful for the nucleophilic astatination of arylboronates (Berdal et al. 2021) and gold nanoparticles (Liu et al. 2021) and is expected to be applied to the production and quality control of various ^211^At-labeled drugs in the future.

## Conclusion

In this study, we have established a stable method for the manufacturing and quality control of [^211^At]NaAt solutions that can be administered intravenously to patients as an investigational product. The solutions were approved by the Institutional Review Board at Osaka University Hospital as an investigational drug for investigator-initiated clinical trials. Our lab was confirmed to be a GMP-compliant manufacturing facility for investigational products through an audit by the National Institutes for Quantum Science and Technology. We are now conducting a phase I clinical trial in patients with refractory differentiated thyroid cancer to evaluate safety and preliminary efficacy. After we finished the current clinical trial, we will move on to the next phase clinical trial to evaluate efficacy with a larger number of patients.

## Data Availability

The datasets used and/or analyzed in the current study are available from the corresponding author upon reasonable request.

## Abbreviations

NIS: sodium-iodine symporter
EOS: end of synthesis
HPLC: high-performance liquid chromatography
ICP-MS: inductively coupled plasma mass spectrometry
LC-MS: liquid chromatography-mass spectrometry
RT: retention time
